# Improving our understanding of the disproportionate incidence of STIs in heterosexual-identifying people of black Caribbean heritage: findings from a longitudinal study of sexual health clinic attendees in England

**DOI:** 10.1136/sextrans-2020-054784

**Published:** 2021-01-29

**Authors:** Megan Bardsley, Sonali Wayal, Paula Blomquist, Hamish Mohammed, Catherine H Mercer, Gwenda Hughes

**Affiliations:** 1 Health Protection Research Unit in Blood Borne and Sexually Transmitted Infections at University College London, in partnership with Public Health England and in collaboration with London School of Hygiene & Tropical Medicine, National Institute for Health Research, London, UK; 2 National Infection Service, Blood Safety, Hepatitis & HIV Division, Public Health England, London, UK; 3 Centre for Population Research in Sexual Health and HIV, University College London, London, UK

**Keywords:** bacterial infections, epidemiology, public health, risk factors, sexual health

## Abstract

**Objective:**

In England, people of black minority ethnicities are at elevated risk of STI diagnosis, especially those of black Caribbean (BC) heritage. Understanding the factors that predict STI acquisition in this population is key to inform prevention measures. We examined the differences in predictors of incident STI diagnoses across ethnic groups in people attending sexual health clinics (SHCs).

**Methods:**

Responses from an attitudinal and behavioural survey run in 16 English SHCs (May–September 2016) were linked to routinely collected national surveillance data on bacterial STI or trichomoniasis diagnoses. Cox proportional hazards models investigated the relationship between participant characteristics and rate of incident STI in the 18 months after survey completion for all heterosexual participants (N=2940) and separately for heterosexual BC (N=484) and white British/Irish (WBI, N=1052) participants.

**Results:**

We observed an overall STI incidence of 5.7 per 100 person-years (95% CI 5.1 to 6.5). STI incidence was higher in participants of BC ethnicity (BC, 12.1 per 100 person-years, 95% CI 9.7 to 15.1; WBI, 3.2 per 100 person-years, 95% CI 2.4 to 4.2), even in adjusted analysis (BC adjusted HR (aHR), 2.60, p<0.001, compared with WBI). In models stratified by ethnicity, having had two or more previous STI episodes in the past year was the strongest predictor of incident STI for both BC (aHR 5.81, p<0.001, compared with no previous episodes) and WBI (aHR 29.9, p<0.001) participants. Aside from younger age (aHR 0.96 for increasing age in years, p=0.04), we found no unique predictors of incident STI for BC participants.

**Conclusions:**

Incident STI diagnoses among SHC attendees in England were considerably higher in study participants of BC ethnicity, but we found no unique clinical, attitudinal or behavioural predictors explaining the disproportionate risk. STI prevention efforts for people of BC ethnicity should be intensified and should include tailored public health messaging to address sexual health inequalities in this underserved population.

## Introduction

In England, almost half a million STI diagnoses are made annually, and this number is rising, particularly for gonorrhoea and syphilis.^1^ Ethnic inequalities in sexual health are well described in England,^2–8^ with people of certain black minority ethnicities, especially those of black Caribbean (BC) heritage, experiencing the highest STI diagnosis rates.^2^ For example, in 2018, the rate of gonorrhoea diagnoses was seven times higher in men of BC ethnicity compared with men of white ethnicity.^9^

Despite this inequality, there are limited data on the attitudes, behaviours and contextual factors that influence STI risk for people of BC heritage in the UK, as highlighted in a recent (and the only) systematic review.^2^ Understanding the predictors of incident STIs can help inform prevention measures and prioritise interventions. These are more likely to be successful if tailored to the needs of the target population, as is the case for STI and HIV interventions for men who have sex with men (MSM).^10^ However, prevention measures tailored to the needs of people of BC heritage are lacking.

Here, we combined rich attitudinal and behavioural data from a bespoke survey with data on STI diagnoses in sexual health clinics’ (SHCs) electronic health records to better understand the predictors of incident STI diagnoses in a large sample of SHC attendees in England, and examined how this varied by ethnicity. We aimed to identify predictors of STI acquisition to support the development of tailored interventions for people of BC heritage.

## Methods

### Behavioural survey

A web-based patient survey was developed as part of a bio-behavioural enhanced surveillance tool (BBEST), as previously described.^11^ In brief, between May and September 2016, the survey was offered to people of all ethnicities attending 16 SHCs across England, purposively selected for relatively high proportions of BC clinic attendees. Eligible participants were aged ≥15 years who reported having sex in the previous 12 months. Participants completed the survey in clinic or elsewhere on personal mobile devices.

### Clinical data

We linked participants’ survey data, with consent, to an extract of their clinical data from the GUMCAD STI Surveillance System (as previously described^11^), which routinely collects patient-level data on STI tests, diagnoses and services from all commissioned sexual health services in England.^12^ All individuals in GUMCAD have a unique clinic-specific identifier, so they can only be followed up within, and not between, SHCs.

A bacterial/protozoal STI diagnosis (hereafter, ‘STI diagnosis’) was defined as a confirmed diagnosis at the study clinic in GUMCAD of any bacterial or protozoal STI, including chlamydia, gonorrhoea, infectious syphilis (primary, secondary and early latent stages) and trichomoniasis. The full list can be found here.^13^

A diagnosis or episode of the time of the survey was defined as having an STI diagnosis on the survey attendance date or in the surrounding ±42 days. Forty-two days is the standard episode length used in GUMCAD analyses to avoid any double counting of STI tests or diagnoses.^14 15^ A sensitivity analysis confirmed that reducing the episode length to 28 or 14 days has negligible impact on episode counts (online supplemental tables 1 and 2).

10.1136/sextrans-2020-054784.supp1 Supplementary data

A previous STI episode was defined as an STI diagnosis within any of the 42-day-long intervals, commencing from the 42 days prior to the survey attendance and going back 365 days.

### Inclusion criteria

Analyses were performed on participants with complete ethnicity information, who identified as male or female and who reported being heterosexual. Women who have sex with women exclusively and MSM were excluded.

### Independent variables

Independent variables were drawn from previous literature and building on the ecosocial and intersectionality theories as described by Wayal *et al*.^8^

Gender, sexual orientation and ethnicity existed in both GUMCAD and the BBEST survey. Survey information was taken as the ‘gold standard’ as they were self-completed at the time of the survey (instead of at registration at the SHC) but substituted from GUMCAD if missing. In both the survey and GUMCAD, participants self-reported their ethnicity from a predefined, standardised list used by all National Health Service providers.^16^

Attitudes to concurrency were measured using a 10-item, 6-point Likert scale^17^ where 1 indicated ‘strongly disagree’ and 6 indicated ‘strongly agree’. A binary variable was created treating scores of 10–30 as negative attitudes and >30 as positive attitudes (the median score was 16). Contraception was based on the participants’ self-reported use of the following methods by themselves or together with their partner in the previous 12 months: any barrier method (*male or female condoms*, *diaphragm*, *spermicide*); permanent/long-acting reversible/hormonal contraception with no use of barrier method (*sterilisation*, *vasectomy*, *implant*, *hormonal or non-hormonal IUDs*, *the pill*, *injection*, *patch*); emergency/natural/no method (‘*morning after pill*’, *emergency IUD*, *abstinence*, *withdrawal*, *natural family planning*).

The participant’s lower-level super output area (LSOA) of residence (LSOA—a geospatial unit used for reporting small area statistics with an average population size of 1614^10^) sourced from GUMCAD was mapped to the 2015 Index of Multiple Deprivation (IMD—an official measure of relative deprivation for small areas of England^10^). The IMD score was grouped into quintiles, with 1 containing the most and 5 containing the least deprived areas.

To investigate how STI risk varied by an individual’s prior diagnosis history, we analysed GUMCAD data from the year before completing the BBEST survey for participants who attended the study clinic during this time. Three summary clinical independent variables were generated:

1. Type (if any) of previous STI diagnosis (three categories): none, chlamydia only and non-chlamydial infection (±chlamydial infection). Chlamydia was separated from other STIs due to its relatively high prevalence.
2. Time since last STI diagnosis (four categories): never in the past year, at survey attendance, ≤6 months before survey and 7–12 months before the survey.
3. Number of previous STI episodes (four categories): no previous episodes, one episode at survey only, one previous episode (±1 at survey) and ≥2 previous episodes (±1 at survey).

### Statistical analyses

The length of time after survey attendance until occurrence of another STI diagnosis at the study clinic was estimated from Kaplan-Meier failure curves. Participants became at risk 43 days/after 6 weeks after the survey attendance and were censored at the end of the study period (548 days/18 months after becoming at risk).

Three Cox regression models determined risk of incident STI diagnosis for (1) participants of all ethnicities and separately for (2) participants of self-defined BC ethnicity (hereon 'BC participants’ for brevity) and (3) participants of self-defined white British/Irish (WBI) ethnicity (hereon ‘WBI participants’ for brevity). For each model, bivariable regression was used to estimate HRs for STI diagnosis by dependent variables.

Multivariable Cox regression was used to determine risk factors for incident STI diagnosis after adjustment for confounders. Variables significant at bivariable level (p<0.05) or of importance a priori (age and gender) were added into multivariable models. Exceptions were collinear pairs (Pearson’s correlation coefficient>0.5, where only one variable was entered) and variables with significant (>10%) missing data (individually tested in multivariable models and likelihood ratio tests used to assess significance).

Age was treated as a continuous variable, indicated by Akaike information criterion(AIC) scores testing non-nested models. A global test of the proportional hazards assumption was performed using Schoenfeld residuals. A formal test of interaction of ethnic group (BC vs WBI) with other variables in model 1 (all ethnicities) was performed with Wald tests.

## Results

### Study characteristics

**Figure 1:**
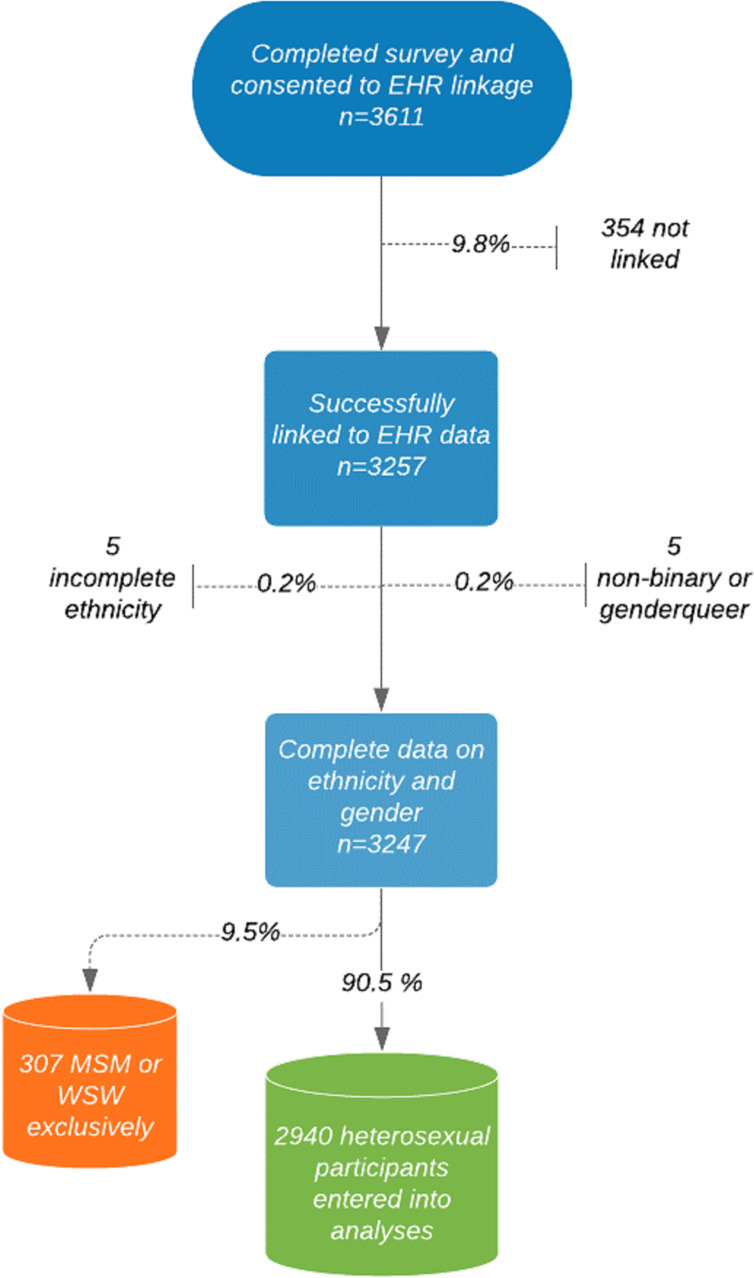
Data management process. EHR, electronic health record; MSM, men who have sex with men; WSW, women who have sex with women.

A total of 3986 participants completed the survey between May and September 2016; 3611 (90.6%) consented to linkage to GUMCAD, of which linkage was achieved for 3257 (90.2%). Of the 3257 participants, 317 (9.7%) were excluded due to sexual orientation or missing data (figure 1).

The majority of the 2940 participants remaining in the analyses were ≥25 years of age (59.2%) and were women (66.8%) (table 1). Almost three-quarters (72.6%) were in employment when completing the survey, while a similar proportion (70.0%) lived in the two most deprived IMD quintiles. Under one-fifth (484/2940, 16.5%) were BC participants and one-third (1052/2940, 35.8%) were WBI participants.

**Table 1:** Sample characteristics of the 2940 survey participants and of those diagnosed with an STI in the 18 months after survey completion

Variable	Total sample characteristics	Outcome: STI diagnosis in follow-up period
	n % (column)	n % (row)
**Demographic**		
Age (years)		
25+ (max. 80)	1739 (59.2)	119 (6.8)
15–24	1201 (40.9)	121 (10.1)
Gender		
Male	975 (33.2)	92 (9.4)
Female	1965 (66.8)	148 (7.5)
IMD quintile		
1 (higher deprivation)	1211 (41.4)	121 (10.0)
2	839 (28.6)	75 (8.9)
3	501 (17.1)	33 (0.4)
4	249 (8.5)	7 (0.3)
5 (lower deprivation)	129 (4.4)	4 (0.7)
Ethnic group		
White British/Irish	1052 (35.8)	49 (4.7)
White other	381 (13.0)	23 (6.0)
Black African	324 (11.0)	29 (9.0)
Black Caribbean	484 (16.5)	79 (16.3)
Black other	35 (1.2)	6 (17.1)
Mixed/other	376 (12.8)	38 (10.1)
Asian	288 (9.8)	16 (5.6)
Degree-level education		
No	1493 (51.3)	160 (10.7)
Yes	1420 (48.8)	79 (5.6)
Currently employed		
No	801 (27.4)	76 (9.5)
Yes	2120 (72.6)	164 (7.7)
**Behavioural and attitudinal**		
Contraception use in p12m (self or partner)		
Any barrier method	1793 (62.4)	143 (8.0)
Permanent/LARC/hormonal (without barrier)	636 (22.1)	48 (7.6)
Emergency/natural/no method only	446 (15.5)	40 (9.0)
Early sexual debut (<16 years of age)		
No	1272 (43.3)	128 (10.1)
Yes	1668 (56.7)	112 (6.7)
Self-perceived risk		
Don't think I'm at risk of getting any STI	1348 (54.2)	133 (9.9)
Think I'm at risk of getting STIs	1139 (45.8)	72 (6.3)
Attitudes to concurrency		
Negative attitude	2517 (90.6)	181 (7.2)
Positive attitude	262 (9.4)	38 (14.5)
Number of partners in p12m		
1	1072 (37.1)	56 (5.2)
2–4	1212 (41.9)	95 (7.8)
5 or more	609 (21.1)	84 (13.8)
Number of new partners in p12m		
None	871 (30.4)	51 (5.9)
1	754 (26.3)	60 (8.0)
2–4	849 (29.6)	72 (8.5)
5 or more	396 (13.8)	49 (12.4)
Partner concurrency in p12m		
No	1944 (69.9)	124 (6.4)
Yes	837 (30.1)	95 (11.4)
Age mixing with any of most recent three partners		
No	1648 (63.6)	133 (8.1)
Yes	942 (36.4)	81 (8.6)
Ethnic mixing with any of most recent three partners		
No	1249 (47.7)	81 (6.5)
Yes	1369 (52.3)	136 (9.9)
Current partnership type		
Married/committed only	1350 (46.4)	80 (5.9)
Casual only	934 (32.1)	97 (10.4)
Both	97 (3.3)	14 (14.4)
Don't have partner	527 (18.1)	46 (8.7)
Clinical*		
Time since last STI diagnosis		
Never	2306 (78.4)	117 (5.1)
At survey attendance	454 (15.4)	91 (20.0)
Within the 6 months before survey	106 (3.6)	16 (15.1)
Within the 7–12 months before survey	74 (2.5)	16 (21.6)
Types of STI previously diagnosed		
None	2306 (78.4)	117 (5.1)
CT only	282 (9.6)	36 (12.8)
Any other STI (including or excluding CT)	352 (12.0)	87 (24.7)
Number of previous STI episodes		
None	2306 (78.4)	117 (5.1)
1 at survey only	376 (12.8)	62 (16.5)
1 episode in p12m (±1 at survey)	230 (7.8)	48 (20.9)
≥2 episodes in p12m (±1 at survey)	28 (1.0)	13 (46.4)

Missing data: IMD (11), degree-level education (27), current employment (19), contraception use (65), self-perceived risk (453), attitudes to concurrency (161), number of partners in p12m (47), number of new partners in p12m (70), partner concurrency in p12m (159), age mixing (350), ethnic mixing (322) and current partnership type (32).

*The time frame for clinical data was the survey attendance (±6 weeks) and the p12m.

IMD, Index of Multiple Deprivation; LARC, long-acting reversible contraception; p12m, previous 12 months.

A total of 634 participants (21.6%) had a clinical record of a bacterial/protozoal STI diagnosis at the survey attendance or in the previous year.

### Model 1 (all ethnic groups): STI incidence and risk factors

#### Bivariable

**Figure 2:**
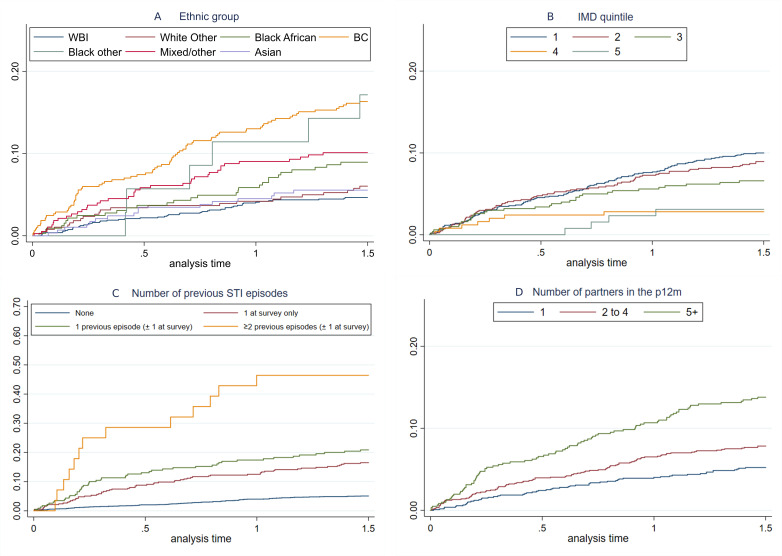
(A–D) Kaplan-Meier failure curves for time (in years) after survey attendance until STI diagnosis. (A) Ethnic group, (B) IMD quintile of residence, (C) number of previous STI episodes, (D) number of partners in the past 12 months. Note: y-axis scales vary. Log-rank tests for all variables p<0.001. BC, black Caribbean; IMD, Index of Multiple Deprivation; WBI, white British/Irish.

The overall incidence of STI diagnosis in the 18 months following survey completion was 5.7 per 100 person-years (95% CI 5.1 to 6.5, data not shown), with 240 (8.2%) participants having at least one diagnosis during this period. Most variables showed a significant association (p<0.05) with a subsequent STI diagnosis, most notably IMD of residence, ethnic group, number of previous STI episodes and number of previous partners (table 2 and figure 2A–D).

**Table 2:** Risk of incident STI diagnosis in the 18 months following survey completion; results from univariable and multivariable Cox regression models, by ethnic group

Variable	Model 1: full study population (N=2940)								Model 2: BC (N=519)					Model 3: WBI (N=1052)				
	Outcome statistics								Outcome statistics					Outcome statistics				
	(STI diagnosis in follow-up period)								(STI diagnosis in follow-up period)					(STI diagnosis in follow-up period)				
	Diagnoses (n)	Person-years	Incidence rate (%)	Univariable		Multivariable			Univariable		Multivariable			Univariable		Multivariable		
				HR	P value	aHR	SE	P value	HR	P value	aHR	SE	P value	HR	P value	aHR	SE	P value
**Demographic**																		
Age (years)*																		
25+ (max. 80)	119	2503	4.8	–	–	–	–	–	–	–	–	–	–	–	–	–	–	–
15–24	121	1686	7.2	**0.96**	<0.01	**0.98**	0.01	0.04	**0.96**	0.01	**0.96**	0.02	0.04	**1.34**	0.31	**0.96**	0.02	0.10
Gender																		
Male	92	1372	6.7	–	–	–	–	–	–	–	–	–	–	–	–	–	–	–
Female	148	2816	5.3	**0.79**	0.07	**0.89**	0.13	0.42	**0.65**	0.07	**0.68**	0.20	0.18	**0.61**	0.08	**0.60**	0.20	0.13
IMD																		
Three least deprived quintiles	44	1274	3.5	–	–	–	–	–	–	–	–	–	–	–	–	–	–	–
Two most deprived quintiles	196	2898	6.8	**1.95**	<0.01	**1.46**	0.25	0.03	**0.69**	0.12	**0.50**	0.14	0.01	**2.63**	0.01	**2.32**	0.84	0.02
Ethnic group																		
WBI	49	1500	3.2	–	–	–	–	–										
White other	23	551	4.2	**1.31**	0.29	**1.40**	0.36	0.18										
Black African	29	462	6.3	**1.95**	<0.01	**1.67**	0.41	0.04										
BC	79	651	12.1	**3.74**	<0.01	**2.60**	0.50	<0.01										
Black other	6	49	12.4	**3.81**	<0.01	**3.57**	1.57	<0.01										
Mixed/other	38	526	7.2	**2.23**	<0.01	**1.69**	0.38	0.02										
Asian	16	417	3.8	**1.20**	0.53	**1.34**	0.39	0.32										
Degree-level education																		
No	160	2095	7.6	–	–	–	–	–	–	–				–	–	–	–	–
Yes	79	2055	3.8	**0.51**	<0.01	**0.74**	0.11	0.04	**0.97**	0.91				**0.45**	0.01	**0.54**	0.17	0.05
Currently employed																		
No	76	1130	6.7	–	–				–	–				–	–			
Yes	164	3031	5.4	**0.81**	0.12				**0.65**	0.07				**0.90**	0.76			
**Behavioural and attitudinal**																		
Contraception use in p12m (self or partner)																		
Any barrier method	143	2561	5.6	–	–				–	–				–	–			
Permanent/LARC/hormonal (without barrier)	40	630	6.3	**0.95**	0.74				**0.67**	0.15				**0.75**	0.47			
Emergency/natural/no method only	48	909	5.3	**1.14**	0.48				**0.56**	0.09				**1.50**	0.30			
Early sexual debut (<16 years of age)																		
No	112	2396	4.7	–	–	–	–	–	–	–				–	–			
Yes	128	1793	7.1	**1.52**	<0.01	**1.13**	0.16	0.40	**1.34**	0.20				**1.73**	0.06			
Self-perceived risk																		
Don't think I'm at risk of getting any STI	133	1893	7.0	–	–				–	–				–	–			
Think I'm at risk of STIs	72	1647	4.4	**0.63**	<0.01				**0.69**	0.143				**0.69**	0.28			
Attitudes to concurrency																		
Negative attitude	181	3600	5.0	–	–				–	–	–	–	–	–	–	–	–	–
Positive attitude	38	355	10.7	**2.12**	<0.01				**1.94**	0.04	**1.25**	0.45	0.54	**3.06**	<0.01	**2.06**	0.81	0.07
Number of partners in p12m																		
1	56	1559	3.6	–	–	–	–	–	–	–	–	–	–	–	–	–	–	–
2–4	95	1730	5.5	**1.52**	0.01	**1.27**	0.23	0.18	**1.92**	0.03	**1.43**	0.49	0.29	**2.53**	0.03	**2.71**	1.35	0.05
5 or more	84	836	10.1	**2.77**	<0.01	**1.97**	0.40	<0.01	**3.23**	<0.01	**1.78**	0.75	0.17	**4.21**	<0.01	**2.46**	1.32	0.09
Number of new partners in p12m																		
None	51	1264	4.0	–	–				–	–				–	–			
1	60	1079	5.6	**1.37**	0.10				**1.53**	0.16				**1.50**	0.42			
2–4	72	1203	6.0	**1.48**	0.03				**1.49**	0.20				**1.70**	0.25			
5 or more	49	547	9.0	**2.20**	<0.01				**3.24**	<0.01				**4.01**	<0.01			
Partner concurrency in p12m																		
No	124	2805	4.4	–	–				–	–				–	–			
Yes	95	1166	8.2	**1.83**	<0.01				**1.78**	0.01				**1.99**	0.02			
Age mixing with any of most recent three partners																		
No	133	2346	5.7	–	–				–	–				–	–	–	–	–
Yes	81	1341	6.0	**1.06**	0.66				**0.61**	0.07				**1.73**	0.06	**2.37**	0.78	0.01
Ethnic mixing with any of most recent three partners																		
No	81	1800	4.5	–	–									–	–			
Yes	136	1923	7.1	**1.56**	<0.01				**1.20**	0.45				**1.54**	0.16			
Current partnership type																		
Married/committed only	80	1952	4.1	–	–	–	–	–	–	–	–	–	–	–	–			
Casual only	97	1309	7.4	**1.80**	<0.01	**1.29**	0.22	0.13	**2.15**	<0.01	**1.44**	0.44	0.23	**1.98**	0.05			
Both	14	132	10.6	**2.55**	0.01	**1.65**	0.50	0.10	**1.88**	0.25	**1.14**	0.65	0.81	**2.69**	0.19			
Don't have partner	46	751	6.1	**1.49**	0.03	**1.30**	0.25	0.17	**1.10**	0.81	**0.65**	0.29	0.33	**1.98**	0.08			
**Clinical**†																		
Time since last STI diagnosis																		
Never	117	3359	3.5%	–	–				–	–				–	–			
At survey attendance	91	590	15.4	**4.34**	<0.01				**4.04**	<0.01				**3.54**	<0.01			
Within the 6 months before survey	16	141	11.4	**3.23**	<0.01				**2.11**	0.09				**2.23**	0.03			
Within the 7–12 months before survey	16	99	16.2	**4.57**	<0.01				**2.78**	0.02				**4.35**	0.02			
Type of STI diagnosed at survey and p12m																		
None	117	3359	3.5	–	–				–	–				–	–			
CT only	36	388	9.3	**2.64**	<0.01				**2.16**	0.02				**1.79**	0.23			
Any other STI (including or excluding CT)	87	442	19.7	**5.52**	<0.01				**4.34**	<0.01				**4.94**	<0.01			
Number of STI episodes at survey and p12m																		
None	117	3359	3.5	–	–	–	–	–	–	–	–	–	–	–	–	–	–	–
At survey only	62	506	12.3	**3.47**	<0.01	**2.68**	0.44	<0.01	**3.05**	<0.01	**2.65**	0.80	<0.01	**2.34**	0.03	**1.65**	0.65	0.21
1 previous episode (±1 at survey)	48	296	16.2	**4.57**	<0.01	**3.21**	0.58	<0.01	**3.47**	<0.01	**2.64**	0.88	<0.01	**4.26**	<0.01	**3.34**	1.38	<0.01
≥2 previous episodes (±1 at survey)	13	28	46.6	**12.53**	<0.01	**6.85**	2.10	<0.01	**5.54**	<0.01	**5.81**	2.73	<0.01	**28.00**	<0.01	**29.94**	23.92	<0.01

Missing data full population: see table 1 footnote.

Missing data BC: degree-level education (5), current employment (1), contraception use (14), self-perceived risk (76), attitudes to concurrency (27), number of partners in p12m (11), number of new partners in p12m (14), partner concurrency in p12m (28), age mixing (66), ethnic mixing (62) and current partnership type (5).

Missing data WBI: IMD (7), degree-level education (6), current employment (4), contraception use (15), self-perceived risk (142), attitudes to concurrency (43), number of partners in p12m (10), number of new partners in p12m (15), partner concurrency in p12m (35), age mixing (104), ethnic mixing (95) and current partnership type (9).

Bold values indicate statistical significance.

*Age modelled as continuous.

†The time frame for clinical data was the survey attendance (±6 weeks) and the p12m.

aHR, adjusted HR; BC, black Caribbean; IMD, Index of Multiple Deprivation; LARC, long-acting reversible contraception; p12m, previous 12 months; SE, Standard error; WBI, white British/Irish.

#### Multivariable

The number of new partners and concurrent partners in the past year was collinear with the number of partners in the past year. We kept number of partners in the model as this is the more widely used indicator of partner change and had the least missing data. All three clinical variables were collinear; we kept the number of previous STI episodes as this is the most clinically informative in practice. Self-perceived risk of STI and ethnic mixing had high non-response and did not impact results when initially included in the final model, and so were excluded.

After adjustment, six variables were significantly associated (p<0.05) with incident STI diagnosis in the 18-month follow-up period (table 2). ‘Black other’ participants were at highest risk of incident diagnosis compared with WBI participants (adjusted HR (aHR) 3.57, 95% CI 1.50 to 8.45), followed by BC participants (aHR 2.60, 95% CI 1.78 to 3.80). Further analyses were not performed on black other participants due to low numbers in this group (n=35).

In the adjusted model, those who had a past diagnosis were at higher risk than those who did not; the risk of incident diagnosis increased with the number of previous STI episodes (aHR for one previous episode=3.22; ≥2 previous episodes=6.85, compared with no previous episodes). Those living in the two most deprived IMD quintiles had a higher risk of incident infection (aHR 1.46) than those in the three least deprived quintiles.

Tests for interaction suggested that IMD and number of previous STI episodes had differing effects on subsequent diagnosis risk in BC compared with WBI participants. Separate Cox models were therefore run on the 484 BC participants and 1052 WBI participants.

### Model 2 (BC only): STI incidence and risk factors

#### Bivariable

The incidence of STI diagnosis among BC participants was 12.1 per 100 person-years (95% CI 9.7 to 15.1), with 16.3% being diagnosed in the follow-up period (table 1). Variables that showed significant bivariable associations with subsequent STI diagnosis were age, attitudes toward concurrency, partner number, number of new partners, partner concurrency, current partnership type and all three clinical variables (table 2).

#### Multivariable

IMD of residence was entered into multivariable modelling despite non-significance in bivariable analyses due to effect modification between the BC and WBI groups (identified in model 1). In the adjusted model, three variables were significantly associated with subsequent STI diagnosis for BC participants: age (aHR 0.96, p=0.04), IMD of residence (two most deprived quintiles, aHR 0.50, p=0.01) and previous STI episodes (≥2 previous episodes, aHR 5.81, p<0.01).

### Model 3 (WBI only): STI incidence and risk factors

#### Bivariable

The incidence of STI diagnosis among WBI participants was 3.2 per 100 person-years (95% CI 2.4 to 4.2), with 4.7% being diagnosed in the follow-up period (table 1). Ten variables showed significant bivariable associations with subsequent STI diagnosis (table 2).

#### Multivariable

Age mixing was retained in the multivariable model despite high non-response (10%), as indicated by a likelihood ratio test. In the adjusted WBI model, five variables remained significantly associated with subsequent STI diagnosis (table 2). Significant effects remained for IMD and education, while effect estimates were weakened for attitudes toward currency and reported partner numbers. Age mixing conferred 2.4 times the risk of a subsequent STI diagnosis (this effect was increased after adjustment). Previous STI diagnosis remained the strongest predictor of subsequent diagnosis (≥2 previous episodes, aHR 29.94, p<0.01).

### Differences in STI risk factors between model 2 (BC) and model 3 (WBI)

#### Bivariable

There were several differences between the BC and the WBI models. Notably, living in deprived areas was not associated with subsequent diagnosis for BC participants (HR 0.69, p=0.16) but was for WBI participants (HR 2.63, p=0.01). Degree-level education did not reduce the risk of a subsequent STI diagnosis for BC participants (HR 0.97, p=0.91) but did so for WBI participants (HR 0.45, p=0.01). Increasing age was associated with a reduced risk of subsequent diagnosis for BC participants (HR 0.96, p<0.001) but not for WBI participants (HR 1.34, p=0.31).

#### Multivariable

For several factors such as IMD, attitudes to concurrency, partner numbers and previous STI episodes, adjusted effect estimates were generally lower for BC than for WBI (the aHR for ≥2 previous STI episodes was very high for WBI). Increasing age imparted a small reduction in risk of subsequent STI diagnosis for BC participants (aHR 0.96, p=0.04) but not for WBI (aHR 0.96, p=0.1). IMD of residence became a significant predictor of subsequent STI diagnosis for BC participants after adjustment (those in the most deprived areas had almost half the rate of STI as those in the least deprived areas (aHR 0.50, p=0.01), in contrast to the WBI model where the reverse was seen (aHR 2.32, p=0.02, table 2).

We hypothesised that the reduced incidence of STI among BC participants (relative to WBI) living in deprived areas might be explained by geographical variability in SHC access. However, when we repeated the modelling process, including only BC and WBI participants who reattended their clinic in the study period, effect estimates for the most deprived IMD quintiles remained the same for both groups (BC aHR 0.42, p=0.03; WBI aHR 2.05, p=0.04; data not shown).

## Discussion

We linked rich attitudinal and behavioural survey data with longitudinal clinical records of SHC attendees in England to explore unique predictors of incident STI diagnoses in study participants of BC heritage. We found that participants of BC ethnicity experienced over 3-fold higher incidence of STI than participants of WBI ethnicity. Greater risk of STI was associated with previous STI diagnosis, positive attitudes to, and engaging in, concurrent partnerships and greater partner numbers among participants of BC ethnicity, but this was also the case for participants of WBI ethnicity. Greater risk of STI was associated with younger age for BC only, but there were no clinical, attitudinal or behavioural predictors of increased risk unique to BC participants in adjusted analyses.

To our knowledge, we are the first to use rich survey data on attitudes and contexts to examine ethnic differences in predictors of incident STI for SHC attendees in England. Other studies reporting increased risk of STI among BC people have not performed risk factor comparisons across ethnic groups.^2–4 6^ A strength of this study is the design; by selecting SHCs with a relatively large proportion of BC attendees, and with a sample size of almost 3000 participants, we could perform detailed analyses stratified by ethnic group. Moreover, STI diagnoses were clinic-verified, reducing reporting bias in the outcome. However, we acknowledge several limitations. First, we cannot draw inferences to the wider population of all SHCs attendees or the non-clinic attending population. We were also unable to consider all sexual identities due to low numbers of participants who identified as MSM or women who have sex with women exclusively. Second, participants of black other ethnic group exhibited the highest rates of STI compared with WBI; however, separate analyses on this group was not possible due to small numbers limiting statistical power. Statistical power remains limited in the model with BC-only participants due to the small number diagnosed with an STI in the study period (n=79), and additionally, we recognise that this still may be a heterogeneous group.^18^ Third, we may underestimate incidence as tracking patient attendances at other clinics is not possible in GUMCAD. However, we expect most participants to reattend the same clinic (at least in our 18-month time frame).^19^ Lastly, while we included a wide range of variables, item non-response meant several potentially important factors such as ethnic mixing and self-perceived risk of infection were not included in multivariable modelling. Moreover, there was no variable on condom use available from the survey that could have been used as a risk factor independently from the use of condoms in the past year in the context of contraception. However, as the target population was those identifying as heterosexual, then we assume that this question would have been perceived as relevant by participants included in this analysis, and as the lookback period corresponds to the entire year (eg, vs condom use at last sex with a particular partner), we consider this to be a reasonable measure of condom use.

Disproportionately high rates of bacterial STIs among black compared with white ethnic groups in England have been reported for decades, and it is concerning that this inequality persists.^7 20^ Across all SHC attendees, and in common with other studies,^5 6 8 21^ STI incidence was associated with younger age, previous STI diagnosis, living in more deprived areas and other behavioural factors such as multiple, concurrent partnerships. Even after accounting for these characteristics, participants of BC heritage still experienced two and a half times greater incidence of STI diagnoses than WBI participants, in accordance with a systematic review and national probability surveys that failed to find specific determinants to fully explain ethnic differences in sexual health outcomes in England.^2 3 8^

It is likely that the sustained high rates of STIs experienced by people of BC heritage reflect a complex interplay between broader structural determinants of health and their influence on individual-level and sexual network factors,^22 23^ none of which was measured here. The background prevalence of untreated infection within sexual networks will influence risk even when there is little absolute difference in risk behaviours of network members. Improved characterisation of transmission networks is needed to identify optimal approaches for interventions.

While there were no behavioural, attitudinal or clinical characteristics that uniquely predicted increased STI risk for BC attendees, previous STI diagnosis was the strongest indicator of subsequent infection for both BC and WBI participants. This underlines the importance of questions on STI history as part of a risk assessment and, potentially, for triaging into a more intensive intervention pathway.^24^ While there were some shared predictors of incident diagnoses between ethnic groups, the effect sizes of these predictors were generally lower for BC relative to WBI participants. This suggests that for BC participants, sexual network effects may have a greater influence on STI acquisition than individual-level risk factors (compared with WBI participants), consistent with previous findings.^3^

Interestingly, younger age did predict STI risk in BC but not in WBI participants. The National Chlamydia Screening Programme offers opportunistic screening to all sexually active young people in England and, between 2015 and 2019, there was a disproportionate increase in testing among non-white ethnic groups and positivity was highest among those of black ethnicity.^25^ If young BC people accessed chlamydia screening through SHCs, this could have increased the likelihood of an STI diagnosis. Further analyses are needed to explore this relationship further. The reduced incidence of STIs among BC participants living in the most deprived areas was unexpected and was not simply an artefact of unequal access to sexual health services, and contrasts with previous studies showing increased risk for black minority ethnicities living in the most socioeconomically deprived areas of England.^5 26^ However, the effect was not observed in bivariable analysis, suggesting some confounding and/or a chance observation.

Our study highlights the continued disproportionately high STI incidence among people of BC heritage. STI prevention efforts should be intensified and should include tailored public health messaging to address this considerable health inequality. Further study into the broader determinants of risk and characteristics of transmission networks is vital to design bespoke interventions for BC communities and to address sexual health inequalities in this underserved population.

**Key messages**

- We investigated ethnic differences in the incidence and predictors of STI diagnoses, focusing on people of black Caribbean (BC) heritage due to their disproportionate STI risk.
- BC participants had almost threefold higher STI incidence compared with white British/Irish participants, even after adjustment for demographic, clinical, attitudinal and behavioural characteristics.
- Previous STI diagnosis/es indicated future diagnosis for both ethnic groups, with no clinical, attitudinal or behavioural predictors of risk unique to BC participants.
- STI prevention efforts for people of BC heritage should be intensified and should include tailored public health messaging to address this considerable health inequality.

## Data Availability

Data are available upon reasonable request. The data that support the findings of this study are available from University College London on reasonable request and with permission of Public Health England.
