# Supplementary material for: Improving our understanding of the disproportionate incidence of STIs in heterosexual-identifying people of black Caribbean heritage: findings from a longitudinal study of sexual health clinic attendees in England
Source: Sex Transm Infect. 2021 Jan 29;98(1):23–31. doi: 10.1136/sextrans-2020-054784 (PMC8785042; doi:10.1136/sextrans-2020-054784)
Supplement: Supplementary data [file sextrans-2020-054784supp001.pdf]

Supplementary tables

Supplementary table 1: Number of STI tests and diagnoses in sexual health services in England, presented by sexual orientation, year, and episode length used in GUMCAD cleaning (42 days or 28 days)

	2012			2013			2014			2015			2016		
	42 days	28 days	% difference	42 days	28 days	% difference	42 days	28 days	% difference	42 days	28 days	% difference	42 days	28 days	% difference
**All attendees**															
CT diagnoses	108,620	109,173	**0.5%**	117,512	118,049	**0.5%**	120,973	121,641	**0.6%**	118,550	119,133	**0.5%**	124,593	125,204	**0.0%**
CT tests	1,407,941	1,413,134	**0.4%**	1,523,663	1,530,606	**0.5%**	1,612,587	1,622,572	**0.6%**	1,650,131	1,660,697	**0.6%**	1,694,675	1,706,375	**0.7%**
Gonorrhoea diagnoses	26,880	27,046	**0.6%**	31,145	31,314	**0.5%**	37,116	37,431	**0.8%**	41,262	41,584	**0.8%**	36,244	36,471	**0.6%**
Gonorrhoea tests	1,378,635	1,393,712	**1.1%**	1,491,434	1,508,654	**1.2%**	1,580,378	1,601,010	**1.3%**	1,617,049	1,639,461	**1.4%**	1,654,781	1,678,694	**1.4%**
Syphilis diagnoses	3,001	3,011	**0.3%**	3,310	3,327	**0.5%**	4,401	4,412	**0.2%**	5,281	5,294	**0.2%**	5,920	5,935	**0.3%**
Syphilis tests	1,029,937	1,035,243	**0.5%**	1,101,973	1,108,007	**0.5%**	1,167,218	1,174,890	**0.7%**	1,201,335	1,210,648	**0.8%**	1,242,507	1,253,490	**0.9%**
HIV diagnoses	4,209	4,220	**0.3%**	4,149	4,154	**0.1%**	4,246	4,250	**0.1%**	3,746	3,748	**0.1%**	3,101	3,101	**0.0%**
HIV tests	1,079,863	1,087,183	**0.7%**	1,153,144	1,161,622	**0.7%**	1,209,958	1,220,309	**0.9%**	1,228,533	1,240,270	**1.0%**	1,255,121	1,267,971	**1.0%**
**MSM**															
CT diagnoses	8,416	8,484	**0.8%**	9,536	9,602	**0.7%**	11,891	11,982	**0.8%**	12,828	12,917	**0.7%**	12,900	12,980	**0.6%**
CT tests	97,874	99,998	**2.2%**	112,737	115,249	**2.2%**	142,637	146,908	**3.0%**	164,085	169,768	**3.5%**	173,413	179,295	**3.4%**
Gonorrhoea diagnoses	10,933	11,041	**1.0%**	14,009	14,122	**0.8%**	18,575	18,782	**1.1%**	22,419	22,625	**0.9%**	17,584	17,708	**0.7%**
Gonorrhoea tests	97,524	99,621	**2.2%**	112,378	115,071	**2.4%**	142,421	146,754	**3.0%**	163,855	169,586	**3.5%**	172,820	178,710	**3.4%**
Syphilis diagnoses	2,138	2,144	**0.3%**	2,410	2,425	**0.6%**	3,526	3,535	**0.3%**	4,185	4,194	**0.2%**	4,788	4,802	**0.3%**
Syphilis tests	83,787	84,795	**1.2%**	96,503	97,833	**1.4%**	122,760	124,947	**1.8%**	141,822	144,837	**2.1%**	153,502	157,056	**2.3%**
HIV diagnoses	2,016	2,020	**0.2%**	2,089	2,090	**0.0%**	2,321	2,324	**0.1%**	2,046	2,048	**0.1%**	1,570	1,570	**0.0%**
HIV tests	89,980	91,829	**2.1%**	102,126	104,500	**2.3%**	122,912	126,245	**2.7%**	137,928	142,132	**3.0%**	143,889	148,336	**3.1%**
**Women and heterosexual men**															
CT diagnoses	100,204	100,689	**0.5%**	107,976	108,447	**0.4%**	109,082	109,659	**0.5%**	105,722	106,216	**0.5%**	111,693	112,224	**0.5%**
CT tests	1,310,067	1,313,136	**0.2%**	1,410,926	1,415,357	**0.3%**	1,469,950	1,475,664	**0.4%**	1,486,046	1,490,929	**0.3%**	1,521,262	1,527,080	**0.4%**
Gonorrhoea diagnoses	14,492	14,540	**0.3%**	15,983	16,032	**0.3%**	17,189	17,285	**0.6%**	17,517	17,626	**0.6%**	17,902	17,996	**0.5%**
Gonorrhoea tests	1,206,773	1,219,035	**1.0%**	1,313,505	1,327,369	**1.1%**	1,365,800	1,381,389	**1.1%**	1,385,609	1,401,634	**1.2%**	1,426,878	1,444,401	**1.2%**
Syphilis diagnoses	750	752	**0.3%**	780	782	**0.3%**	752	754	**0.3%**	876	880	**0.5%**	973	973	**0.0%**
Syphilis tests	894,166	898,202	**0.5%**	962,363	966,869	**0.5%**	1,000,555	1,005,816	**0.5%**	1,021,286	1,027,355	**0.6%**	1,058,995	1,066,292	**0.7%**
HIV diagnoses	1,960	1,966	**0.3%**	1,890	1,893	**0.2%**	1,787	1,788	**0.1%**	1,560	1,560	**0.0%**	1,397	1,397	**0.0%**
HIV tests	933,978	939,088	**0.5%**	1,005,064	1,010,915	**0.6%**	1,040,921	1,047,654	**0.6%**	1,050,394	1,057,653	**0.7%**	1,080,055	1,088,289	**0.8%**

Supplementary table 2: Number of STI tests and diagnoses in sexual health services in England in 2016, presented by sexual orientation and episode length used in GUMCAD cleaning (42 days, 28 days, 14 days).

Diagnoses/tests	2016				
	42 days	28 days	14 days	% difference (42 to 28 days)	% difference (42 to 14 days)
**All**					
CT diagnoses	124,593	125,204	126,634	**0.5%**	**1.6%**
CT tests	1,694,675	1,706,375	1,735,027	**0.7%**	**2.4%**
Gonorrhoea diagnoses	36,244	36,471	36,965	**0.6%**	**2.0%**
Gonorrhoea tests	1,654,781	1,678,694	1,705,021	**1.4%**	**3.0%**
Syphilis diagnoses	5,920	5,935	5,972	**0.3%**	**0.9%**
Syphilis tests	1,242,507	1,253,490	1,262,922	**0.9%**	**1.6%**
HIV diagnoses	3,101	3,101	3,101	**0.0%**	**0.0%**
HIV tests	1,255,121	1,267,971	1,279,576	**1.0%**	**1.9%**
**MSM**					
CT diagnoses	12,900	12,980	13,163	**0.6%**	**2.0%**
CT tests	173,413	179,295	185,710	**3.4%**	**7.1%**
Gonorrhoea diagnoses	17,584	17,708	17,965	**0.7%**	**2.2%**
Gonorrhoea tests	172,820	178,710	185,540	**3.4%**	**7.4%**
Syphilis diagnoses	4,788	4,802	4,829	**0.3%**	**0.9%**
Syphilis tests	153,502	157,056	159,970	**2.3%**	**4.2%**
HIV diagnoses	1,570	1,570	1,570	**0.0%**	**0.0%**
HIV tests	143,889	148,336	152,190	**3.1%**	**5.8%**
**Women and non-MSM men**					
CT diagnoses	111,693	112,224	113,471	**0.5%**	**1.6%**
CT tests	1,521,262	1,527,080	1,549,317	**0.4%**	**1.8%**
Gonorrhoea diagnoses	18,660	18,763	19,000	**0.6%**	**1.8%**
Gonorrhoea tests	1,481,961	1,499,984	1,519,481	**1.2%**	**2.5%**
Syphilis diagnoses	1,132	1,133	1,143	**0.1%**	**1.0%**
Syphilis tests	1,089,005	1,096,434	1,102,952	**0.7%**	**1.3%**
HIV diagnoses	1,531	1,531	1,531	**0.0%**	**0.0%**
HIV tests	1,111,232	1,119,635	1,127,386	**0.8%**	**1.5%**
